# Preparation and Stability Study of Graphene/R141b Nanofluids for Heat Transfer Enhancement

**DOI:** 10.3390/nano16211375

**Published:** 2026-10-27

**Authors:** Jianyang Zhou, Guiyu Zhang, Haiqing Liu, Genge Zhang, Qingqing Chang, Caigui Huang, Xiaoping Luo

**Affiliations:** 1 Guangxi Key Laboratory of International Join for China-ASEAN Comprehensive Transportation, Research Center of Intelligent Manufacturing and Virtual Simulation, School of Intelligent Manufacturing, Nanning University, Nanning 530299, China; zhoujianyang@unn.edu.cn (J.Z.);; 2 Nanning Research Institute, Guilin University of Electronic Technology, Nanning 530004, China; 3 School of Mechanical and Automobile Engineering, South China University of Technology, Guangzhou 510640, China

**Keywords:** graphene/R141b, zeta potential, DLS particle size, stability

## Abstract

The instability of nanofluids leads to particle agglomeration and sedimentation, which clog the heat transfer channels and deteriorate the overall performance of heat exchangers. Accordingly, preparing uniformly dispersed and structurally stable nanofluids serves as a fundamental prerequisite for their practical engineering applications. In this study, refrigerant R141b was selected as the base fluid, and graphene/R141b nanofluids were prepared via a two-step method assisted by ultrasonic oscillation. The effects of ultrasonic oscillation time, dosage of surfactant, and nanoparticle mass concentration on the stability of graphene/R141b were systematically investigated, and zeta potential, DLS particle size, Raman and XRD analysis were used to evaluate the stability of graphene/R141b. The experimental results demonstrate that ultrasonic oscillation time and nanoparticle mass concentration both exert significant influences on the stability of graphene/R141b nanofluid. The SDS surfactant can effectively improve the stability of the prepared graphene/R141b nanofluid. In conclusion, under the conditions of adding SDS surfactant and carrying out ultrasonic oscillation within 120–150 min, graphene/R141b with a mass concentration ranging from 0.05% to 0.4% exhibits satisfactory stability, while excessively prolonged sonication (180 min) reduces the stability of the nanofluid, which provides a promising candidate for future validation of heat-transfer-enhancement performance in heat-exchanger applications.

## 1. Introduction

With the rapid development of high-technology equipment such as MEMS, LED lighting devices, and biochips, a large number of micro-scale heat transfer problems have become urgent issues to be resolved. Existing studies have shown that flow boiling heat transfer in minichannels can achieve high heat dissipation performance under compact device dimensions, which has attracted wide research interest for high-density thermal management [1]. Scholars have conducted extensive research into the effects of minichannel surface modification, heat exchange channel cross-sectional dimension, and working fluid type on enhanced flow boiling heat transfer [2,3,4,5,6,7,8,9].

Since Choi et al. [10] first proposed the concept of nanofluids in 1995, the application of nanofluids in heat transfer has attracted extensive academic attention [11,12,13]. Zhou et al. prepared Al_2_O_3_/R141b with mass fractions ranging from 0.05 wt% to 0.5 wt% and systematically investigated the influence of nanoparticle concentration on the flow boiling heat transfer coefficient in minichannel heat exchangers. Experimental results showed that when the mass fraction of nanoparticles exceeded 0.1 wt%, the boiling heat transfer coefficient decreased with the increase in nanoparticle concentration. This performance degradation is attributed to the instability of Al_2_O_3_/R141b at high mass fractions during flow boiling. More seriously, the instability of nanofluids leads to particle agglomeration and sedimentation, which clog the heat transfer channels and deteriorate the overall heat exchanger performance [5]. Therefore, the stability of nanorefrigerants has a significant impact on the heat transfer performance of heat exchangers. Existing studies have shown that nanoparticle agglomeration will increase the effective particle size in the refrigerant, and then change the boiling heat transfer characteristics [14,15]. In addition, nanoparticle deposition modifies the surface characteristics of the flow channel (e.g., surface roughness, wettability, and active nucleation site density). Such surface alterations dominate the boiling heat transfer behavior, bubble nucleation and growth dynamics, and further disturb the two-phase flow pattern, which ultimately triggers the pressure instability during boiling [16,17]. Accordingly, investigating the dispersion stability of nanorefrigerants is an essential prerequisite for exploring their heat transfer performance in heat exchangers. At present, there are few studies focusing on graphene-refrigerant-based nanofluids for minichannel flow boiling heat transfer. For both traditional particle-based nanofluids and graphene-based nanofluids applied to minichannel boiling, nanoparticle agglomeration (that is, nanofluid instability) remains an unavoidable core issue in practical applications, which severely limits their practical application in flow boiling heat transfer enhancement [18]. Therefore, research on nanofluid stability is of important scientific significance for promoting the application of nanofluids in boiling heat transfer enhancement.

At present, research on the application of graphene-based nanofluids in the field of heat transfer mainly concentrates on investigating the influence of the type and concentration range of graphene-based refrigerants on heat transfer performance [19,20,21,22]. Existing studies indicate that for both conventional nanofluids and graphene-based nanofluids, particle agglomeration, which refers to the instability of the nanofluid working medium, is still a core issue that is difficult to avoid in practical applications, and this problem severely restricts the practical promotion of such nanofluids in the field of heat transfer enhancement. Accordingly, carrying out systematic research on the stability of nanofluid working fluids has important scientific value for promoting the practical application of nanofluids in the field of heat transfer enhancement. Hwang et al. fabricated nanofluids via a two-step approach and explored the effect of different vibration treatment parameters on nanoparticle size [23]. Xie et al. prepared Al_2_O_3_ nanofluids using a two-step method and analyzed nanofluid stability via the mean design method. Taking zeta potential as the evaluation indicator, they systematically investigated multiple influencing factors, including particle concentration, dispersant type, and oscillation duration, and eventually determined the optimal formulation for preparing stable nanofluids [24]. Liu et al. fabricated stable, non-sedimenting water-based graphene oxide nanofluids using deionized water as the base fluid via ultrasonic oscillation without the addition of any dispersant. They analyzed the stability of the prepared nanofluid via zeta-potential measurement combined with DLVO suspension theory, and revealed the intrinsic mechanism underlying the long-term stability of graphene oxide nanofluids [25].

According to the above literature review, current research on the stability of graphene/R141b nanorefrigerants and the effect of stability on heat transfer performance is still relatively insufficient. The preparation of stable, non-sedimenting nanofluids is a fundamental prerequisite for achieving boiling heat transfer enhancement. In this study, graphene/R141b nanofluids were fabricated via ultrasonic oscillation, adopting R141b refrigerant as the base fluid and graphene nanosheets as the dispersed nanoparticles. Zeta potential. DLS particle size, Raman and XRD analyses were employed to characterize the influences of particle concentration, ultrasonic oscillation time and dispersant on the stability of the graphene/R141b nanorefrigerant. An optimized nanorefrigerant with outstanding stability was obtained, which lays a foundation for subsequent research on flow boiling heat transfer enhancement of graphene/R141b nanofluids.

## 2. Materials and Methods

### 2.1. Reagents and Material Characterization

In this work, R141b refrigerant (1,1-dichloro-1-fluoroethane) was selected as the base fluid for nanofluid fabrication. Under atmospheric pressure and room temperature, R141b remains in the liquid phase, with a density of 1186.700 kg/m^3^. The boiling point at atmospheric pressure is 32 °C, and the molecular weight is 116.95 g/mol. Graphene nanoparticles were selected as the dispersed nanoparticles. The purity of the purchased graphene powder reaches 99.7%. To further optimize the dispersion behavior of graphene nanoparticles in the base fluid, dispersants, specifically sodium dodecyl sulfate (SDS), were introduced during the preparation of the nanofluids. SDS is a common anionic surfactant, which can adsorb on graphene surfaces and introduce negative surface charges to produce electrostatic repulsion and inhibit particle aggregation; its density is 1010.00 kg/m^3^, its boiling point at atmospheric pressure is 32 °C, and its molecular weight is 288.380 g/mol. The dispersants represent electrostatic-dominated stabilization mechanisms. We aim to screen a suitable surfactant for graphene/R141b preparation.

### 2.2. Graphene/r141b Nanofluids Preparation

**Figure 1:**
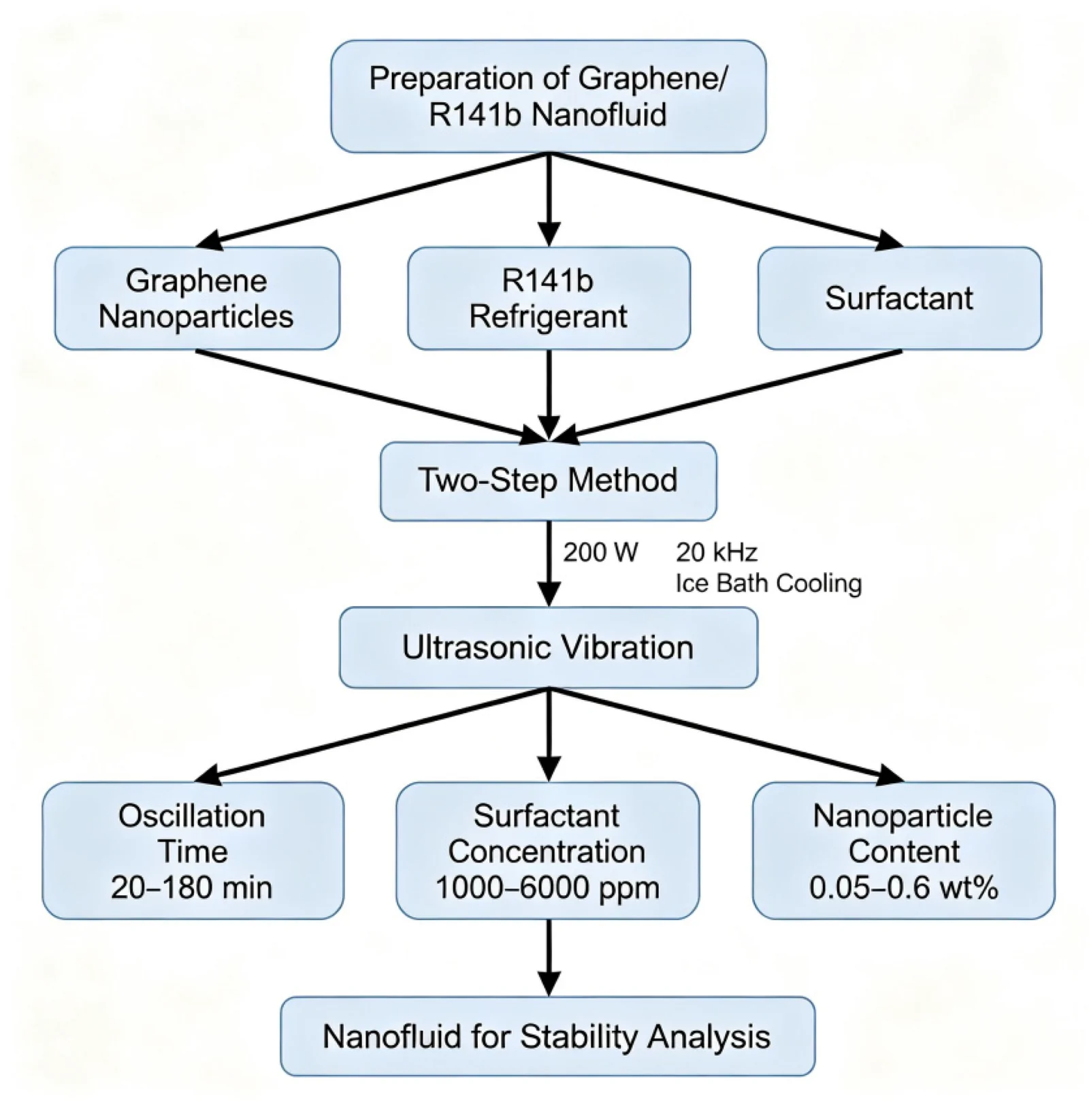
Preparation flowchart of graphene/R141b nanofluids.

In this work, the two-step method was adopted to fabricate nanofluids. Graphene/R141b nanofluid samples with mass fractions ranging from 0.05 wt% to 0.6 wt% were prepared, and the preparation procedure is shown in Figure 1. All samples were prepared with a fixed volume of 40 mL (47.47 g), and the masses of graphene powder and dispersants corresponding to each mass fraction are presented in Table 1. During preparation, an electronic balance with a measurement accuracy of 0.001 mg (100,000-level precision) was utilized to weigh the experimental raw materials, and a 100 mL graduated cylinder with a measurement precision of 1 mL was employed to measure 40 mL (47.47 g) of the R141b base fluid. After the corresponding dispersants were added, the mixture was placed in an ultrasonic oscillator (JY92-IID, Ningbo Scientz Biotechnology Co., Ltd., Ningbo, China). The instrument was operated at an output power of 200 W and fixed frequency of 20 kHz, and the standard duration of ultrasonic treatment was set to 2 h. An ice-water cooling bath was continuously applied during ultrasonication to suppress heat accumulation and reduce the volatilization of volatile R141b refrigerant. The detailed preparation process of graphene/R141b nanofluids is illustrated in Figure 1.

In this research, to investigate the influence of ultrasonic oscillation duration on the stability of graphene/R141b, 0.1 wt% graphene/R141b samples were repeatedly prepared with gradient ultrasonic treatment durations set as 20 min, 40 min, 60 min, 90 min, 120 min, 150 min, and 180 min. Furthermore, to explore the effect of dispersant on the stability of the prepared nanofluids, 0.1 wt% graphene/R141b samples were fabricated by adding SDS, with concentration gradients of the dispersant set as 1000 ppm, 2000 ppm, 3000 ppm, 4000 ppm, 5000 ppm, and 6000 ppm, while all other preparation conditions remained identical.

### 2.3. Physical Properties of Graphene/r141b

The physical property parameters of graphene/R141b in this study can be calculated via the following empirical equations [1,2,3,4,5,26,27], and the physical properties of graphene/R141b are shown in Table 2. It should be noted that these theoretically calculated physical parameters are not adopted for the subsequent stability experiments and data analysis in this work. As shown in Table 2, although the thermal conductivity increases with the increase in nanoparticle mass fraction, the increment of thermal conductivity is very limited. This indicates that the improvement of intrinsic thermal conductivity of graphene/R141b nanofluids has little contribution to the overall heat transfer coefficient of heat exchangers. It further emphasizes that nanofluid stability plays a dominant role in the practical heat exchange performance, which provides a logical premise for the following experimental research on the stability of graphene/R141b nanofluids [5].

(1) $$\varphi_{\mathrm{nf}}=\frac{w\rho_{f}}{(1-w)\rho_{p}+w\rho_{f}}$$

*ρ*_nf_ = (1 − *φ*)*ρ*_f_ + *φρ*_p_ (2)

(3) $$\lambda_{\mathrm{nf}}=\frac{\lambda_{p}+2\lambda_{f}-2(\lambda_{f}-\lambda_{p})\varphi }{\lambda_{p}+2\lambda_{f}+(\lambda_{f}-\lambda_{p})\varphi }\lambda_{f}$$

*μ*_nf_ = *μ*_f_(1 + 2.5*φ* + 6.25*φ*^2^) (4)

*C*_p,nf_ = (1 − *w*)*C*_p,f_ + *wC*_p,p_ (5)

where *φ* denotes the volume fraction, *w* denotes the mass fraction *w* = m_graphene_/(m_graphene_ + m_R141b_)*100%, *ρ* denotes the density, *λ* denotes the thermal conductivity, *μ* denotes the dynamic viscosity, and *C*_p_ denotes the specific heat at constant pressure. The subscript nf denotes nanofluid, the subscript f denotes the base fluid (R141b refrigerant), and the subscript p denotes nanoparticles.

The calculations in Table 2 employed the following graphene properties: the density, specific heat capacity, and thermal conductivity were assumed to be 2200 kg/m^3^, 0.710 kJ/(kg·K), and 3000 W/(m·K), respectively. These values were adopted as modeling assumptions and do not represent measurements of the graphene powder used in this study. The volume fraction was determined from the mass fraction using Equation (1), with both fractions expressed as dimensionless quantities. Specific heat capacity was calculated using the mass-fraction-weighted relation in Equation (5). The contribution of surfactants to the estimated properties was not included. Accordingly, the values in Table 2 are conditional model predictions and should not be regarded as experimentally validated properties of the prepared nanofluids.

## 3. Results and Discussion

### 3.1. Characterization of Graphene Nanoparticles

**Figure 2:**
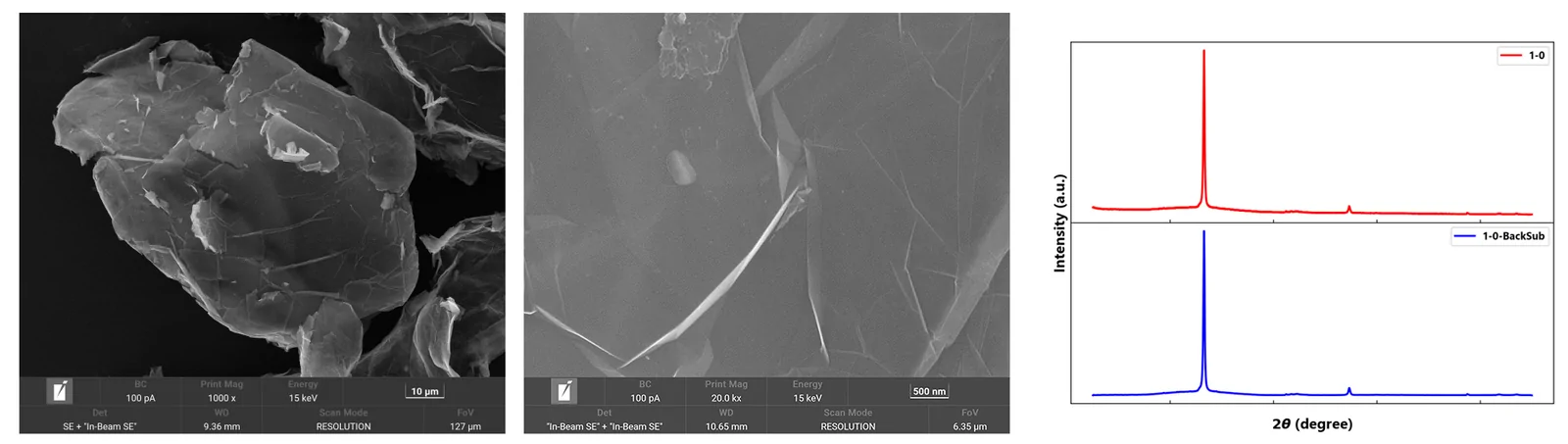
(**a**) SEM image of graphene nanoparticles; (**b**) XRD of graphene nanoparticles.

Figure 2 shows the SEM morphology and XRD pattern of graphene nanoparticles. The XRD pattern presents a sharp characteristic diffraction peak (002) of graphene at 2θ ≈ 26.5°, which corresponds to the stacking structure of graphene sheets. No obvious shift in this characteristic peak is observed, and the peak shape is relatively narrow, indicating that the carbon lattice structure of graphene is well preserved. The SEM image reveals the typical wrinkled sheet structure of graphene with curled edges. The lateral size of the sheets ranges from 10 to 80 μm, and the thickness is no more than 3 nm, which belongs to few-layer graphene. These morphological features are consistent with the sheet stacking information obtained from the XRD pattern, providing a structural basis for the formation of loose and stable clusters in graphene/R141b nanofluids.

### 3.2. Graphene/r141b Nanofluid Stability

#### 3.2.1. Zeta Potential of Graphene/r141b Nanofluid

According to the DLVO theory proposed by Xie et al. [24], particles dispersed within nanofluids are subject to repulsive and attractive potential energies. As inter-particle distance increases, attractive potential gradually dominates. In contrast, shorter inter-particle distances enhance electrostatic repulsion and maintain stable particle dispersion. Nanoparticles induce counter-ion accumulation in the dispersion medium to form an electrical double-layer, and the potential difference across this double-layer is defined as the zeta potential [28]. As a well-accepted quantitative metric for colloidal stability, a larger absolute zeta-potential value indicates stronger electrostatic repulsion and better dispersion stability, while a smaller absolute value suggests a higher tendency for particle aggregation. Therefore, zeta potential was used in this study to characterize the stability of graphene/R141b nanofluids, and the influences of ultrasonic oscillation time, surfactant dosage, and nanoparticle mass fraction were systematically explored. In this work, a Zetasizer Nano ZS90 instrument (Malvern Panalytical, UK) was employed for zeta-potential characterization of graphene/R141b nanofluid. Measurements were performed at 25 °C, which is close to the ambient temperature of our nanofluid preparation experiments. For each sample, repeated measurements were performed. The absolute value of zeta potential was adopted to judge the colloidal stability of graphene/R141b nanofluids. An absolute zeta-potential value above 30 mV was regarded as the criterion for good colloidal stability.

**Figure 3:**
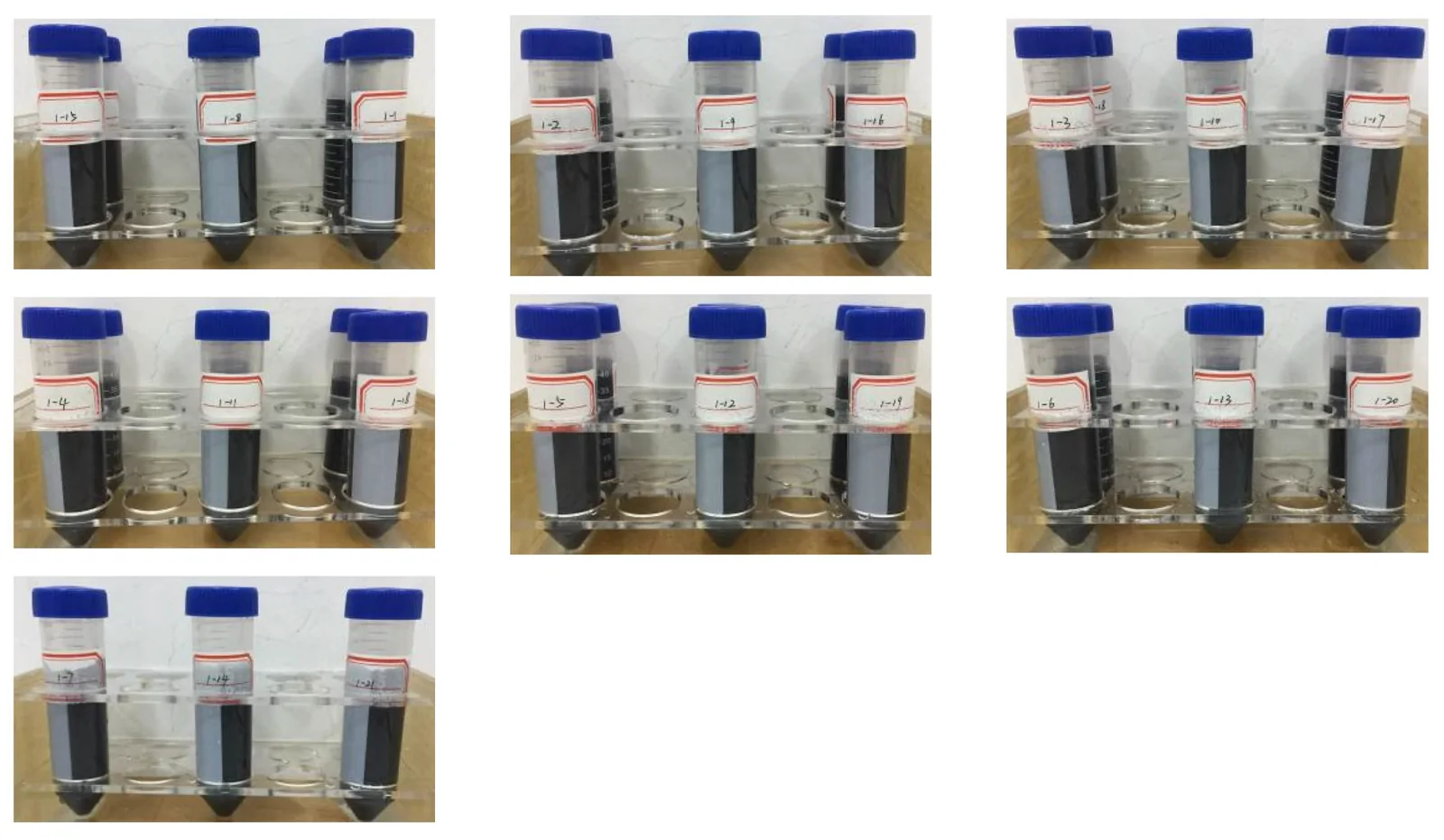
Graphene/R141b under different ultrasonic oscillation times.

Figure 3 shows the 0.1 wt% graphene/R141b nanofluids prepared under ultrasonic oscillation times of 20, 40, 60, 90, 120, 150, and 180 min. Labels 1-1 to 1-7 in the figure correspond to 0.1 wt% graphene/R141b nanofluids prepared without surfactant addition, whereas labels 1-8 to 1-14 represent the 0.1 wt% graphene/R141b nanofluids with 0.1 wt% SDS surfactant added during ultrasonic treatment. It can be observed from Figure 3 that nanoparticles are relatively uniformly dispersed in all samples, and no obvious particle sedimentation occurs. The measurement results are shown in Table 3.

**Figure 4:**
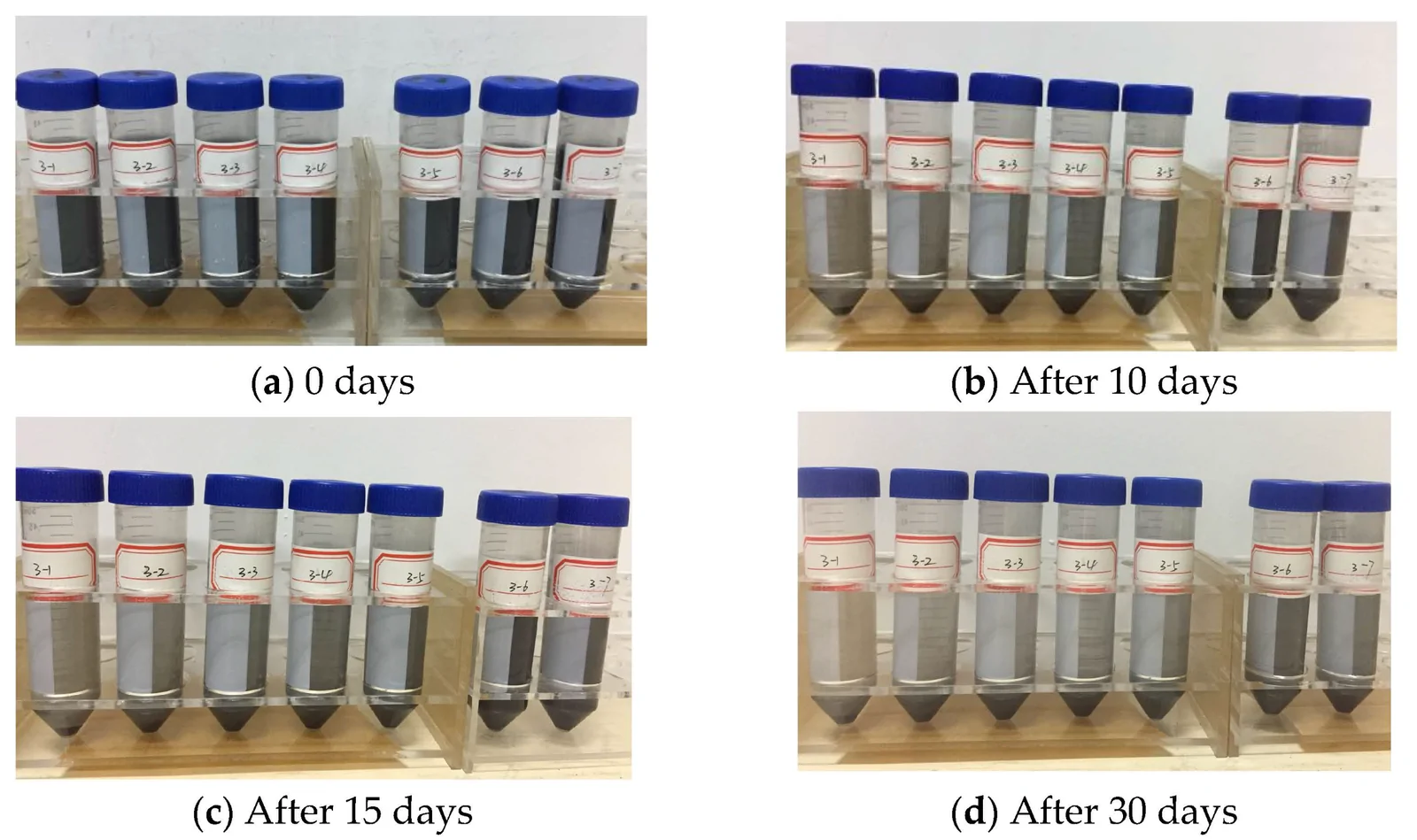
State of nanofluids (0.05 wt% to 0.6 wt% graphene/R141b).

Visual evaluation is a fundamental technique for characterizing the time-dependent stability of nanofluids, which allows intuitive qualitative observation of sedimentation, particle agglomeration, and dispersion homogeneity via monitoring variations in optical properties (including color uniformity, particle dispersion degree, and sediment formation) throughout a predefined observation period. Figure 4 presents the macroscopic morphology of graphene/R141b nanofluid samples (with graphene mass fractions ranging from 0.05 wt% to 0.6 wt%) after 120 min of ultrasonic treatment. At the initial observation time point (day 0), graphene particles are uniformly dispersed in the R141b base solution, with no visible sedimentation observed; meanwhile, the color of the nanofluid gradually deepens as the graphene mass fraction increases. The corresponding zeta-potential characterization results of these samples are summarized in Table 4. Time-resolved static sedimentation photographs (Figure 4) demonstrate that all tested samples maintain a uniformly dispersed black colloidal state at day 0. After 10 days of static storage, slight stratification can be observed in a small number of samples. At the 15-day observation mark, partial sedimentation occurs in samples with non-optimized formulations. After 30 days of static placement, poorly dispersed samples form obvious sediment layers, while the optimized nanofluid formulation still maintains relatively good dispersion homogeneity, with only negligible sedimentation observed. The above time-lapse visualization observations qualitatively reflect the differences in colloidal stability of nanofluids induced by variations in graphene concentration and SDS dosage, which is consistent with the characterization results of DLS particle size and Zeta-potential.

**Figure 5:**
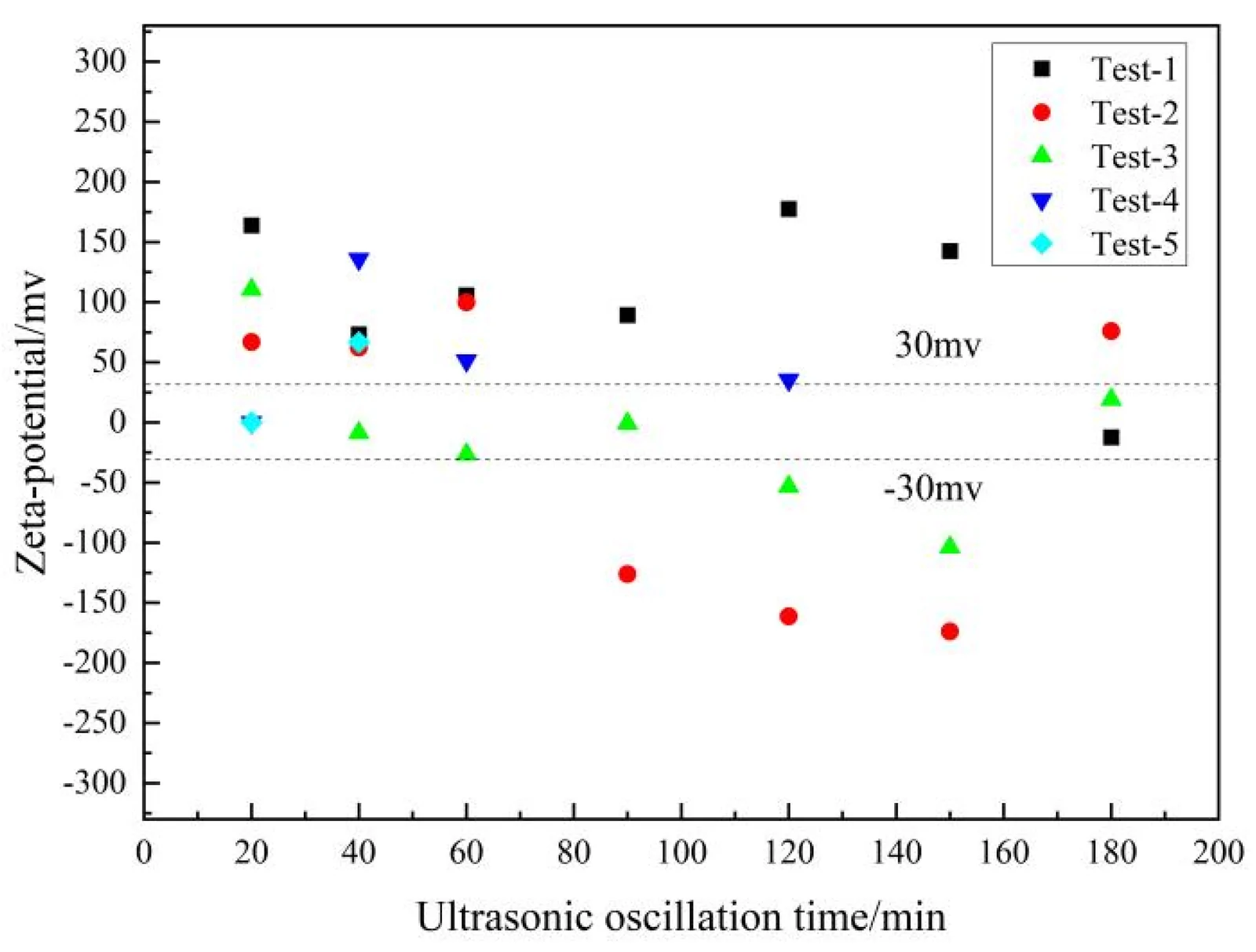
Variation of the zeta potential with oscillation time.

**Figure 6:**
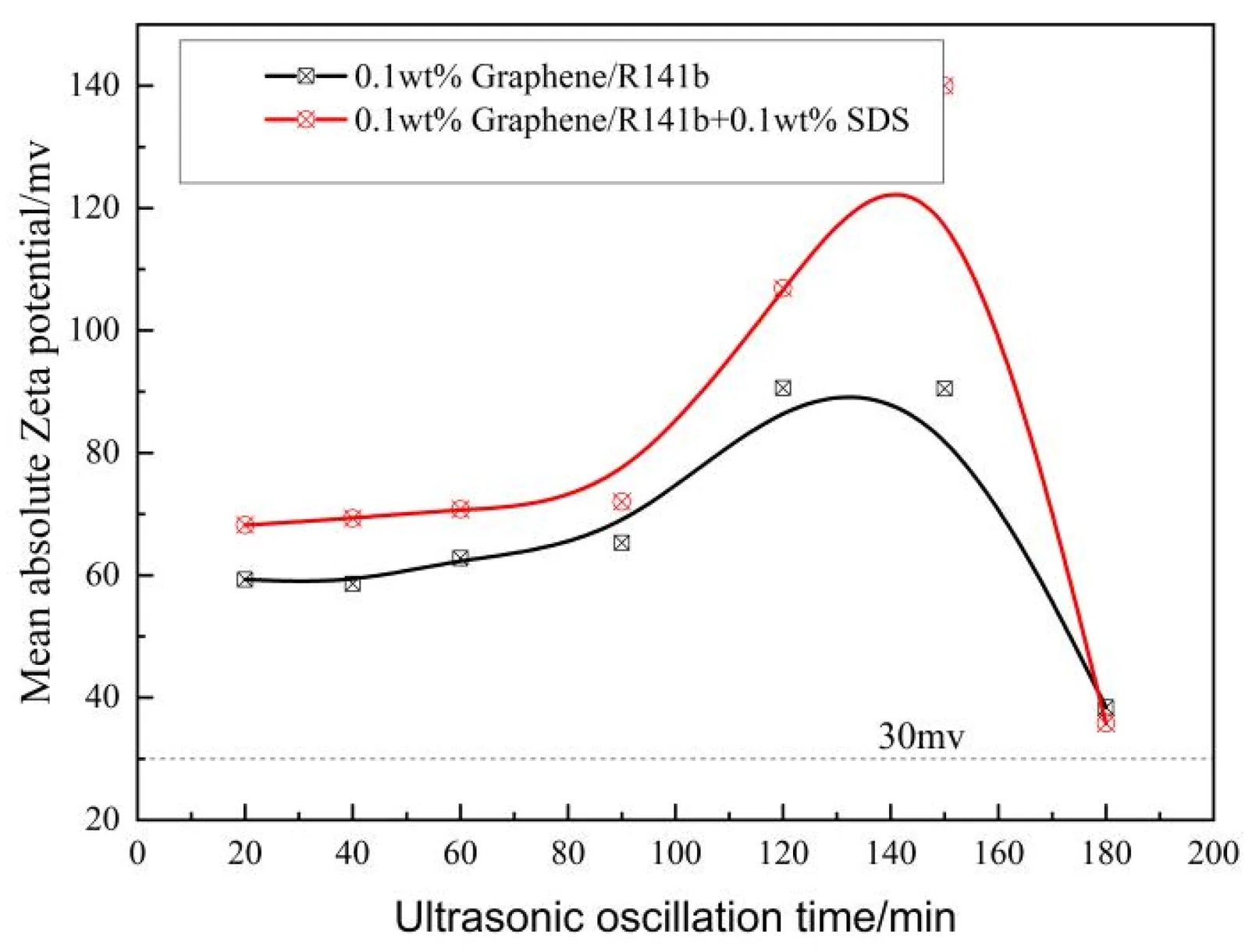
Variation of the zeta potential average value with oscillation time.

Based on the average zeta-potential values calculated from the results in Table 3, for 0.1 wt% graphene/R141b samples prepared with and without SDS dispersant, the average zeta-potential values of both groups exceed 30 mV when the ultrasonic oscillation duration is set to 20 min, which preliminarily indicates that the as-prepared nanofluid systems satisfy the basic stability requirement (see details for Figure 5). However, repeated zeta-potential measurements show that although the average absolute zeta potential meets the stability criterion when ultrasonic oscillation time is longer than 20 min, some individual measurement results still do not reach the stable threshold. When ultrasonic oscillation time is extended to 120 min, all measured absolute zeta-potential values of the nanofluids exceed 30 mV, which verifies that 120 min of ultrasonic oscillation can ensure reliable system stability. When the oscillation duration is further prolonged to 180 min, partial absolute zeta-potential values fall below 30 mV, and the overall average absolute zeta potential shows a gradual downward trend as the oscillation duration increases. Statistical comparison between 120 min and 150 min samples shows no statistically significant difference in mean absolute zeta potential within this optimum window. This phenomenon can be attributed to excessive ultrasonic irradiation: the accumulated heat generated during long-duration ultrasound treatment transfers into the nanofluid system, which in turn damages the dispersion stability of the nanoparticles. Under the same preparation conditions, the average absolute zeta potential of the nanofluids increases after adding the SDS dispersant, which objectively confirms that the addition of dispersant can effectively improve the stability of graphene/R141b nanofluids (see details for Figure 6).

**Figure 7:**
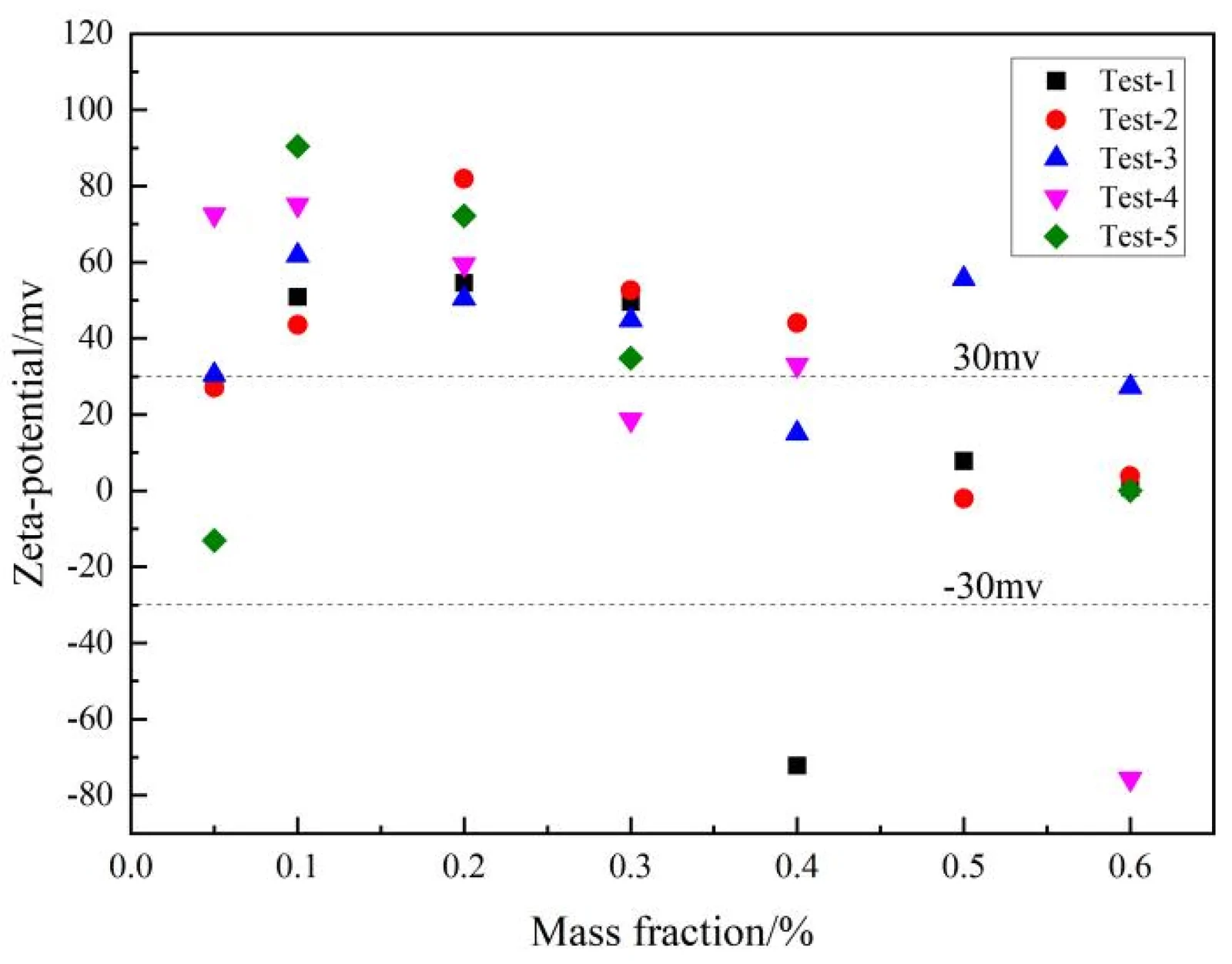
Variation of the average zeta potential with mass fraction.

**Figure 8:**
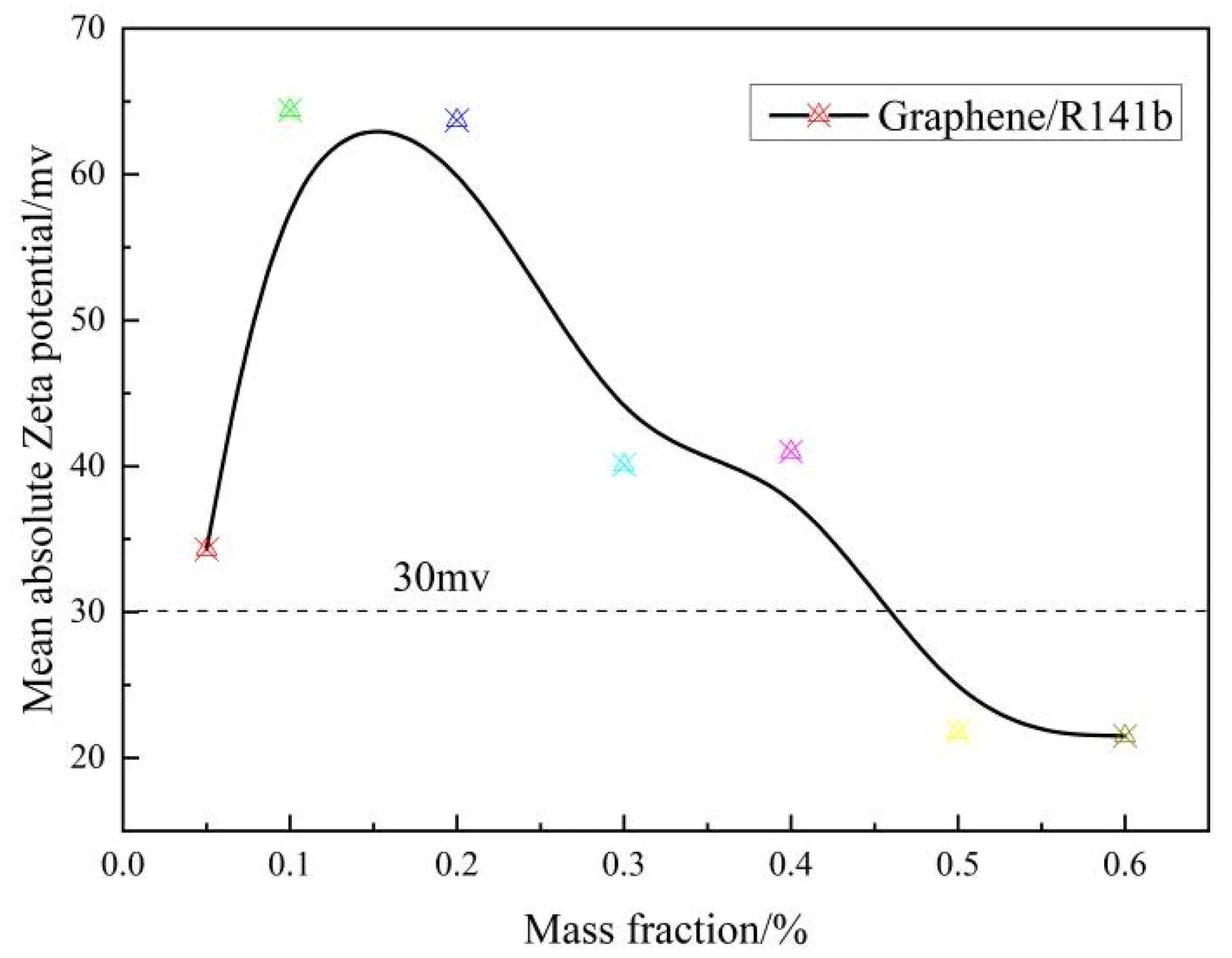
Variation of the average zeta potential with mass fraction.

According to the correlation between zeta potential and system stability, a system exhibits favorable stability when the absolute value of zeta potential exceeds 30 mV (|Zeta-potential| > 30mV). Accordingly, for the graphene/R141b nanorefrigerant prepared by the two-step method, good stability can be maintained within the low-concentration range of 0.05 wt%–0.4 wt%, whereas the stability degrades at higher mass fractions (see details for Figure 7 and Figure 8).

#### 3.2.2. DLS Particle Size of Graphene/r141b Nanofluid

**Figure 9:**
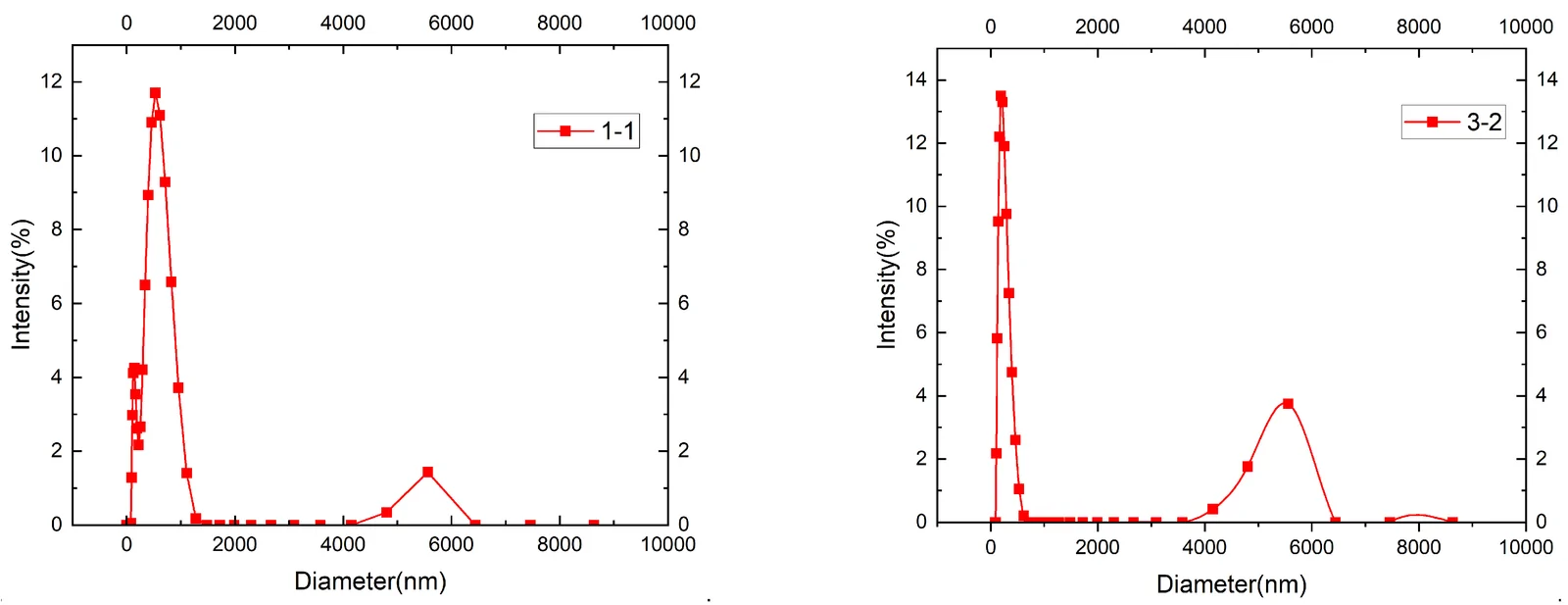
Particle size results of graphene/R141b nanofluids at oscillation time.

Dynamic light scattering (DLS) was employed to characterize the dispersion efficiency of graphene/R141b nanofluids and acquire their particle size distribution. As presented in Figure 9, particle size measurement of all samples was conducted via a Malvern Zetasizer Pro instrument. Comparative analysis was performed between Sample 1-1 (0.1 wt% graphene/R141b with SDS, 20 min of ultrasonic vibration) and Sample 3-2 (0.1 wt% graphene/R141b with SDS, 120 min of ultrasonic vibration). The results indicate that the average particle size of Sample 1-1 is 397 nm, while the average particle size of Sample 3-2 is 259 nm. Statistical analysis of measurement data demonstrates that the average particle size of graphene/R141b nanofluid subjected to 120 min of ultrasonication decreases by 34.7% relative to the sample treated with 20 min of ultrasonication. This finding confirms that prolonging oscillation time can promote the dispersion of graphene nanosheets, which further verifies that oscillation time has a significant effect on the colloidal stability of graphene-based nanofluids.

**Figure 10:**
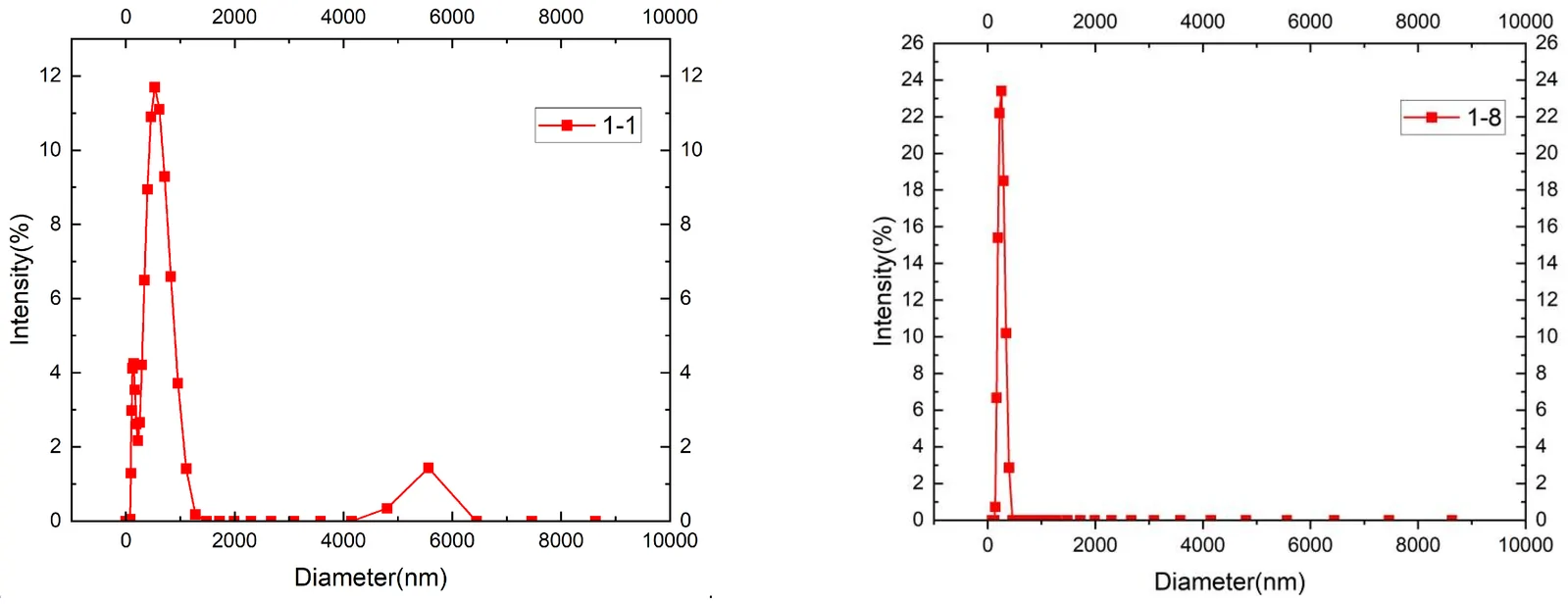
Particle size results of graphene/R141b nanofluids in the presence and absence of SDS.

Figure 10 illustrates the effect of the dispersant sodium dodecyl sulfate (SDS) on the particle size of graphene/R141b nanofluids. Comparative experiments between Sample 1-1 and Sample 1-8 were conducted under identical graphene mass concentration and identical ultrasonication duration. The measurement results indicate that the average particle size of Sample 1-1 is 397 nm, while that of Sample 1-8 decreases to 368 nm, corresponding to a reduction of approximately 7.3% in average particle size. This result confirms that the incorporation of SDS can reduce the apparent particle size of graphene nanosheets, which further verifies that the addition of dispersant exerts a non-negligible effect on the colloidal stability of graphene-based nanofluids.

**Figure 11:**
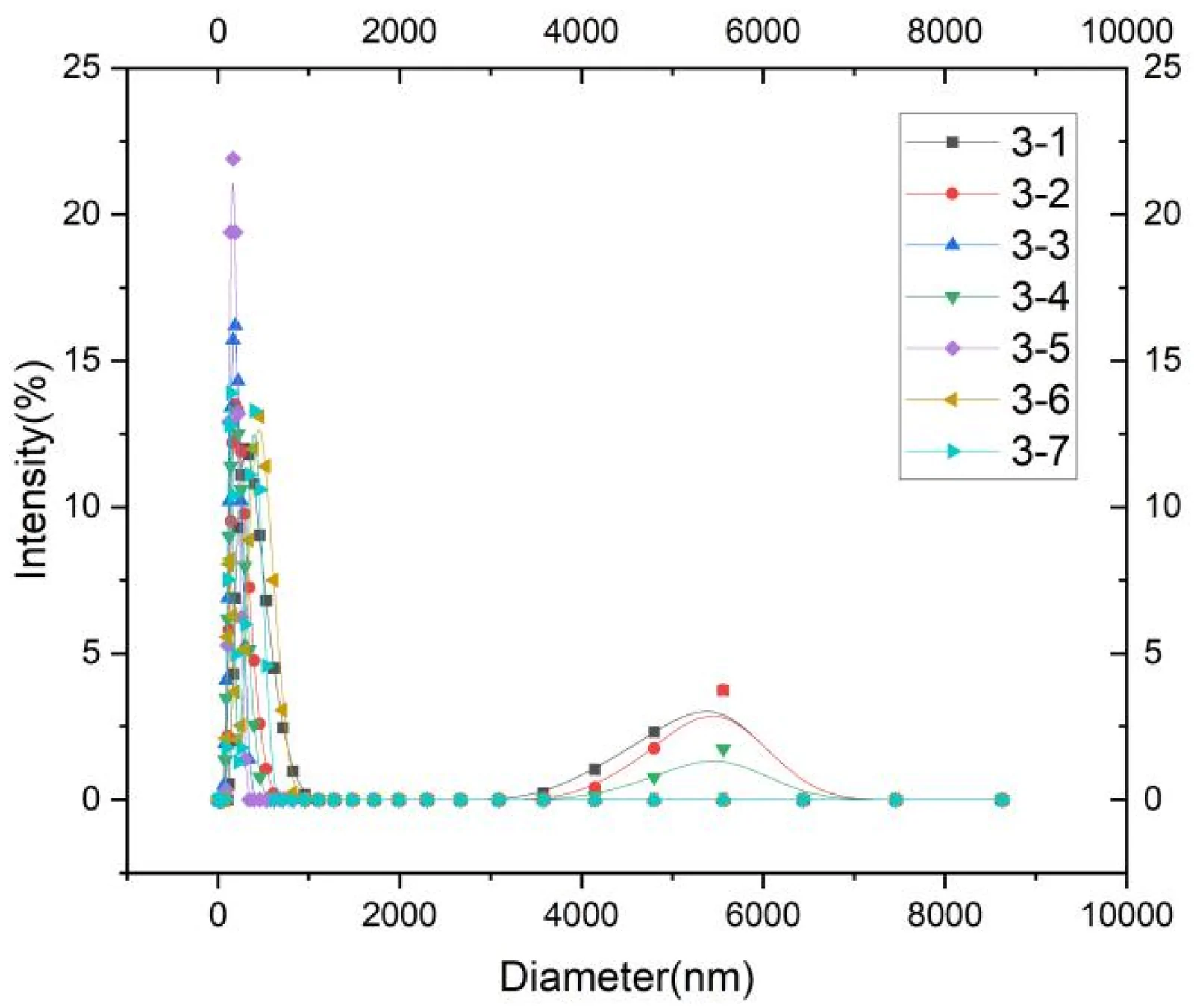
Particle size of graphene/R141b at different mass fractions.

**Figure 12:**
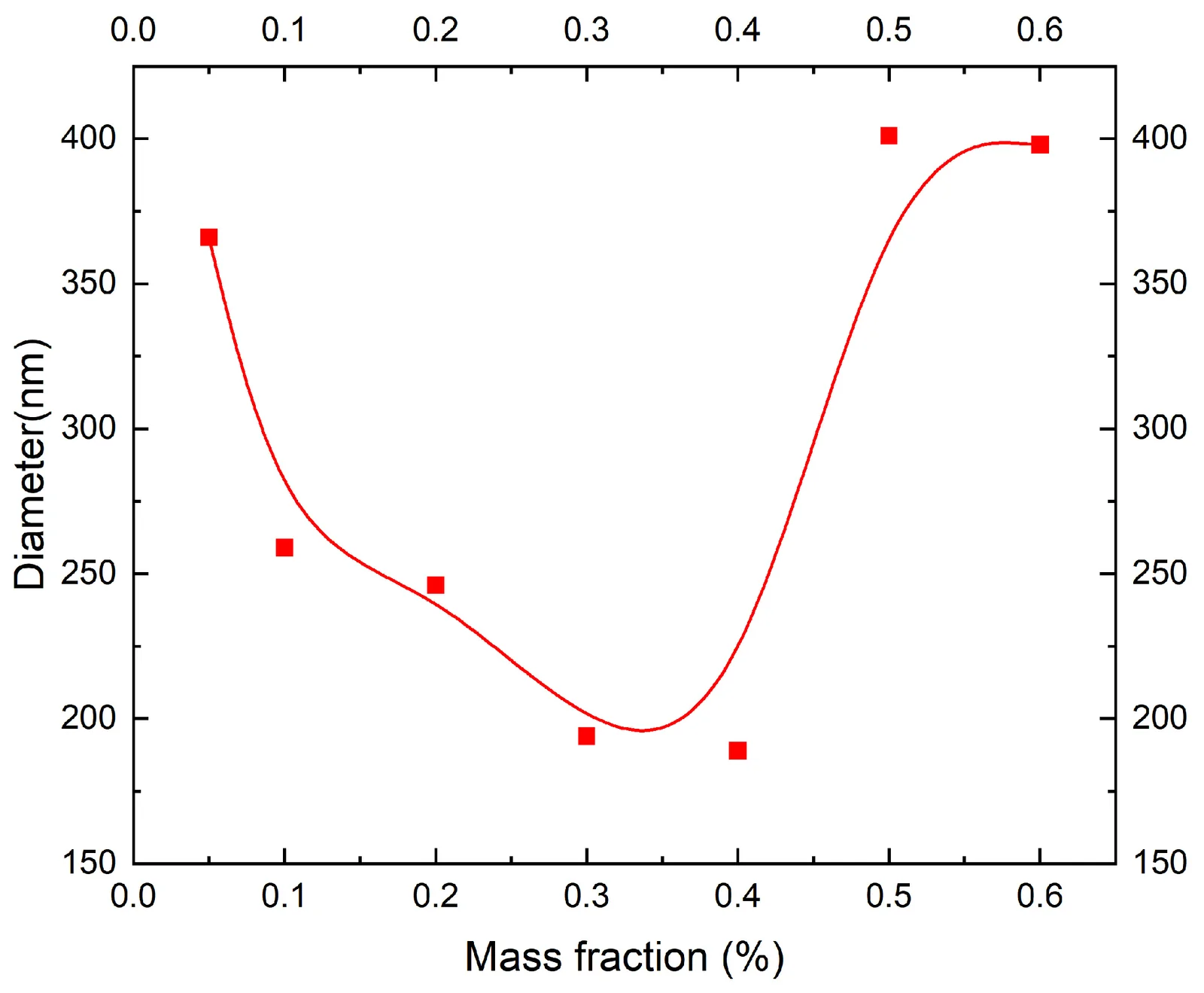
Dependence of graphene/R141b particle size on mass concentration.

Figure 11 and Figure 12 show the particle size measurement results of graphene/R141b nanofluids with different mass fractions (Samples 3-1, 3-2, 3-3, 3-4, 3-5, 3-6 and 3-7) prepared under 120 min ultrasonication with the addition of SDS. The measured particle diameters of nanofluids with mass fractions ranging from 0.05 wt% to 0.6 wt% are 366 nm, 259 nm, 246 nm, 194 nm, 189 nm, 401 nm and 398 nm, respectively. It can be observed that the average particle diameter of the nanofluid shows a nonlinear change with the increase in graphene concentration, and this variation trend is highly consistent with the concentration-dependent trend obtained from zeta-potential measurements. This result further verifies the corresponding relationship between the concentration of graphene nanofluid and its colloidal stability.

#### 3.2.3. Raman and XRD Analysis of Graphene/r141b Nanofluid

**Figure 13:**
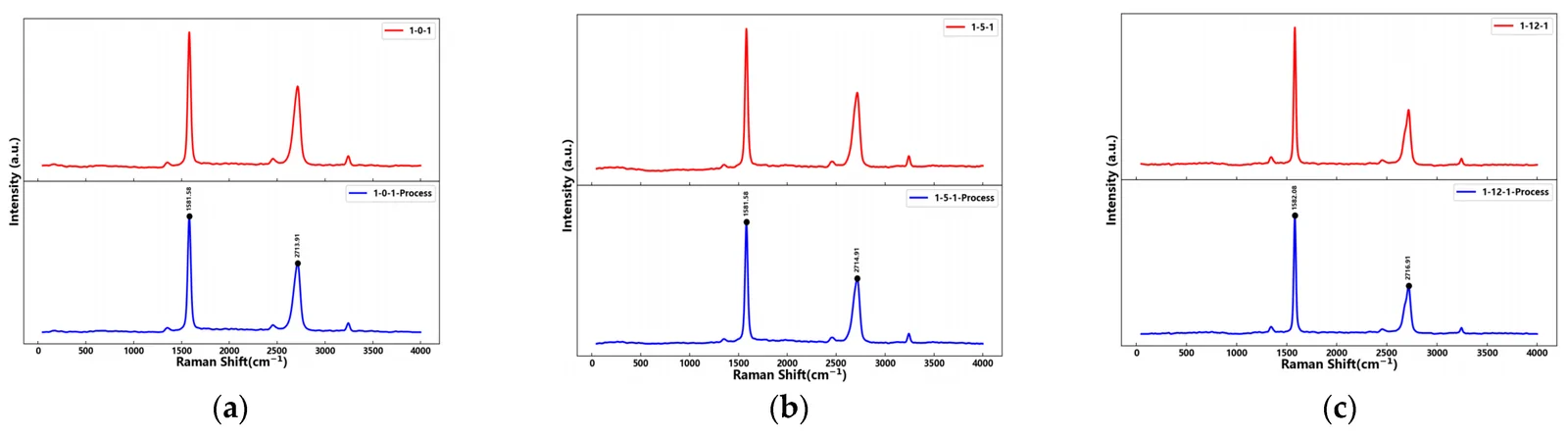
Raman results of samples. (**a**) Graphene nanoparticles; (**b**) graphene/R141b Sample 1-5; (**c**) graphene/R141b Sample 1-12.

Figure 13a shows the Raman spectra of graphene powder, Figure 13b shows the Raman spectra of 0.1 wt% graphene/R141b nanofluid after 120 min ultrasonication, and Figure 13c shows the Raman spectra of 0.1 wt% graphene/R141b nanofluid with 0.1 wt% SDS after 120 min ultrasonication. Raman spectra were recorded at room temperature on a LabRAM HR Evolution spectrometer (HORIBA, Japan). All samples exhibit characteristic D peak (~1350 cm^−1^) and G peak (~1580 cm^−1^) of graphene, as well as a 2D peak near 2700 cm^−1^. The D peak corresponds to structural defects in carbon materials, the G peak originates from the in-plane vibration of sp^2^-hybridized carbon atoms, and the profile and intensity of the 2D peak can be used to evaluate the number of graphene layers. Compared with pristine graphene powder, the nanofluid samples after ultrasonication show a slight increase in the *I_D_/I_G_* ratio, indicating that long-time ultrasonic exfoliation introduces minor edge defects without destroying the intrinsic sp^2^ carbon framework of graphene. The 2D peak maintains a distinct single-band profile, confirming the few-layer nature of graphene, which is consistent with the XRD and SEM results above. By comparing nanofluid samples with and without SDS, no obvious degradation of the *I*_D_*/I*_G_ ratio is observed after adding SDS, and the characteristic 2D peak is well preserved. This demonstrates that the SDS dispersant only provides physical dispersion and does not damage the crystal structure of graphene sheets. Thin graphene nanosheets obtained by ultrasonic exfoliation, combined with the surface modification of SDS, reduce the van der Waals attraction between sheets and inhibit restacking and agglomeration, facilitating the favorable dispersion stability of the nanofluid.

**Figure 14:**
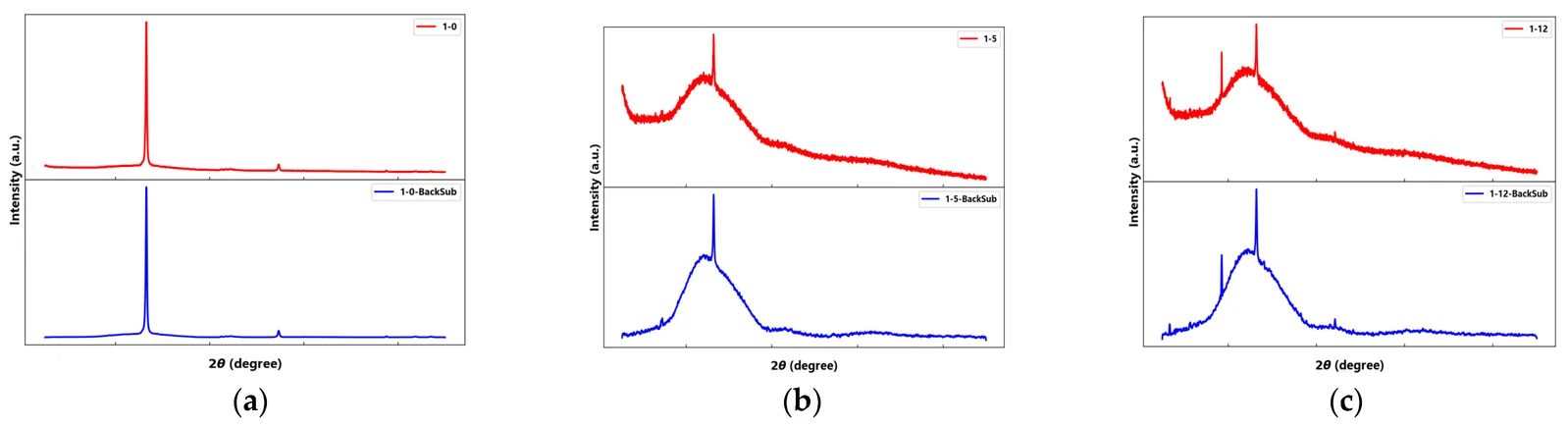
XRD results of samples. (**a**) Graphene nanoparticles; (**b**) graphene/R141b Sample 1-5; (**c**) graphene/R141b Sample 1-12.

Figure 14 presents the XRD patterns of pristine graphene powder, a dried sample from 0.1 wt% graphene/R141b nanofluid after 120 min ultrasonication, and a dried sample from 0.1 wt% graphene/R141b nanofluid containing 0.1 wt% SDS after 120 min ultrasonication. The pristine graphene exhibits a sharp (002) diffraction peak at 2θ ≈ 26.5°, corresponding to the stacking structure of graphene sheets. After 120 min of ultrasonic treatment, the intensity of the graphene (002) diffraction peak decreases significantly and the peak becomes broadened, indicating that prolonged ultrasonic exfoliation effectively reduces the number of stacked layers and the sheet thickness of graphene. With the addition of SDS dispersant, further broadening of the (002) peak and a weaker stacking degree are observed, which suggests that SDS can adsorb onto graphene surfaces and inhibit sheet restacking during drying. These results confirm the synergistic effect of ultrasonic exfoliation and SDS in weakening the van der Waals force between graphene sheets and mitigating sheet agglomeration. This facilitates the stable dispersion of graphene in the R141b working fluid and is consistent with the nanofluid stability characterized by DLS particle size and zeta potential.

### 3.3. ANOVA for the Effects of Various Factors on Nanofluid Stability

Additionally, in the present study, analysis of variance (ANOVA) is introduced to analyze the influence of ultrasonic oscillation time, surfactant addition, and nanoparticle mass concentration on the stability of graphene/R141b. It should be noted that the factor has a significant effect if *F* > *F_a_* (*df_i_*, *df_e_*) = *F*_crit_. The value is calculated using Equations (6)–(16). The analysis results can be found in Table 5 and Table 6; ultrasonic oscillation time and nanoparticle mass concentration have a significant effect on the stability of graphene/R141b. *SS* stands for Sum of Squares, *df* for Degrees of Freedom, *MS* for Mean Square, *F* for F ratio, and *F-Crit* for Critical F-value. For surfactant addition, *p* = 0.069, which is slightly above the conventional 0.05 significance level. Although zeta-potential measurements show observable improvement after SDS addition, this factor cannot be regarded as statistically significant under the current number of replicates.

(6) $$F_{r}=\frac{MS_{r}}{MS_{e}}$$

(7) $$F_{s}=\frac{MS_{s}}{MS_{e}}$$

where *r* and *s* are the level numbers of impact factors. *MS* is the mean square, which is defined by the following calculation formulas.

(8) $$MS_{r}=\frac{SS_{r}}{df_{r}}$$

(9) $$MS_{s}=\frac{SS_{s}}{df_{s}}$$

(10) $$MS_{e}=\frac{SS_{e}}{df_{e}}$$

where *df* and *SS* are the degrees of freedom and the sum of squares of deviation from the mean, which can be defined by the following calculation formulas.

*df_r_* = *r* − 1 (11)

*df_s_* = *s* − 1 (12)

*df_e_* = (*r* − 1)(*s* − 1) (13)

(14) $$SS_{r}={\sum_{j=1}^{s}\sum_{i=1}^{r}\left({\bar{x}}_{i•}-\bar{x}\right)}^{2}=s{\sum_{i=1}^{r}\left({\bar{x}}_{i•}-\bar{x}\right)}^{2}$$

(15) $$SS_{s}={\sum_{i=1}^{r}\sum_{j=1}^{s}\left({\bar{x}}_{•j}-\bar{x}\right)}^{2}=r{\sum_{j=1}^{s}\left({\bar{x}}_{•j}-\bar{x}\right)}^{2}$$

(16) $$SS_{e}={\sum_{i=1}^{r}\sum_{j=1}^{s}\left(x_{\mathrm{ij}}-{\bar{x}}_{i•}-{\bar{x}}_{•j}-\bar{x}\right)}^{2}$$

## 4. Conclusions

In this study, graphene/R141b nanofluids were prepared via a two-step method combined with ultrasonic oscillation, using R141b refrigerant as the base fluid. The effects of ultrasonic oscillation time, surfactant dosage, and nanoparticle mass concentration on the stability of graphene/R141b nanofluids were systematically analyzed with zeta potential, DLS particle size, Raman and XRD as the evaluation indices. The main conclusions are summarized as follows:

(1) Ultrasonic oscillation time and nanoparticle mass concentration exert significant effects on the stability of graphene/R141b nanofluids. Although zeta-potential measurements indicate observable improvement following SDS addition, the effect of surfactant addition cannot be considered statistically significant given the current number of replicates.

(2) The addition of SDS surfactant can effectively improve the stability of the nanofluid. The optimal stability for graphene/R141b nanofluids is achieved within an ultrasonic oscillation duration of 120 min-150 min. Further extension to 180 min reduces stability due to excessive ultrasonic irradiation.

(3) Graphene/R141b nanofluids exhibit excellent stability when the mass concentration of nanoparticles ranges from 0.05 wt% to 0.4 wt%, while the stability of the nanofluids deteriorates as the mass concentration increases to 0.5 wt%–0.6 wt%. This composition range yields graphene/R141b nanofluids with favorable colloidal stability, which serve as promising candidates for subsequent validation of heat-transfer-enhancement performance in heat exchanger tests.

## Figures and Tables

**Table 1:** Mass of raw materials for preparation of graphene/R141b nanofluid.

Mass Fraction/%	R141b/g	Graphene/g	SDS/g
0.05%	47.47	0.024	0.013 × 10^−3^
0.1%	47.47	0.049	0.050 × 10^−3^
0.2%	47.47	0.098	0.202 × 10^−3^
0.3%	47.47	0.147	0.455 × 10^−3^
0.4%	47.47	0.197	0.810 × 10^−3^
0.5%	47.47	0.246	1.268 × 10^−3^
0.6%	47.47	0.295	1.830 × 10^−3^

**Table 2:** Physical properties of graphene/R141b nanofluid.

*w*/%	*φ*/%	*ρ*/kg/m^3^	*C*_p_/kJ/(kg·K)	*λ*/W/(m·K)	*μ* × 10^−3^/Pa·s
R141b	0	1186.700	1.188	0.084000	0.3150
0.05%	0.027	1186.973	1.188	0.084068	0.3152
0.1%	0.054	1187.247	1.188	0.084136	0.3154
0.2%	0.108	1187.794	1.187	0.084272	0.3159
0.3%	0.162	1188.342	1.187	0.084409	0.3163
0.4%	0.216	1188.890	1.186	0.084546	0.3167
0.5%	0.270	1189.439	1.186	0.084683	0.3171
0.6%	0.325	1189.989	1.185	0.084820	0.3176

**Table 3:** Measured zeta potential of graphene/R141b under different ultrasonic oscillation times.

Material Composition	No.	Time	Zeta-Potential/mV										Average Value/mV
			T-1	T-2	T-3	T-4	T-5	T-6	T-7	T-8	T-9	T-10	
0.1 wt% graphene/R141b	1-1	20	−194.1	158.5	16.3	−113.3	−3.3	−1.2	21	8.1	−0.8	−76.9	59.3
	1-2	40	0	0	0	0	34.6	71.4	194.5	159.1	87.3	42	58.6
	1-3	60	76	−39.3	0	128.9	70.1	-	-	-	-	-	62.8
	1-4	90	154.7	83.1	12.3	4.8	6.4	-	-	-			65.3
	1-5	120	−64.5	101.1	162.2	96.7	28.7	-	-	-	-	-	90.6
	1-6	150	193.1	50.4	65.8	143.3	0	-	-	-	-	-	90.5
	1-7	180	77.2	0	0	0	115.1	-	-	-	-	-	38.4
0.1wt% graphene/R141b +0.1%wtSDS	1-8	20	163.9	66.8	110.5	0	0	-	-	-	-	-	68.2
	1-9	40	73.2	62.3	−8.6	135.9	66.7	-	-	-	-	-	69.3
	1-10	60	105.7	99.8	−26.5	51.3	-	-	-	-	-	-	70.8
	1-11	90	89.2	−126.2	−0.8	-	-	-	-	-	-	-	72.0
	1-12	120	177.6	−161.4	−53.3	35.3	-	-	-	-	-	-	106.9
	1-13	150	142.5	−173.9	−103.7	-	-	-	-	-	-	-	140.0
	1-14	180	−12.4	75.9	19.1	-	-	-	-	-	-	-	35.8

**Table 4:** Measured zeta potential of graphene/R141b with different nanoparticle mass fractions.

*w*/%	No.	Zeta-Potential Test/mV					Average Value/mV
		Test-1	Test-2	Test-3	Test-4	Test-5	
0.05%	3-1	28.1	27.1	30.5	72.5	−13.1	34.3
0.1%	3-2	50.9	43.6	61.8	75.1	90.5	64.4
0.2%	3-3	54.6	81.9	50.5	59.5	72.2	63.7
0.3%	3-4	49.6	52.6	44.8	18.6	34.8	40.1
0.4%	3-5	−72.2	44.0	15.1	33.0	-	41.0
0.5%	3-6	7.8	−2.1	55.7	-	-	21.8
0.6%	3-7	0.8	3.8	27.3	−75.6	0	21.5

**Table 5:** ANOVA results of the influences of ultrasonic oscillation time and dispersant on nanofluid stability.

Source	SS	df	MS	F	*p*-Value	F-Crit	Significance
Ultrasonic oscillation time	7920.5	6	1320.1	9.5	7.5 × 10^−3^	4.28	*
Dispersant dosage	679.0	1	679.0	4.9	6.9 × 10^−2^	5.99	
Error	833.6	6	138.9				
Total	9433.1	13					

**Table 6:** ANOVA results for the effects of nanoparticle mass fraction on nanofluid stability.

Source	*SS*	*df*	*MS*	*F*	*p-Value*	*Fcrit*	Significance
Between-group	22,269.5	6	3711.6	4.05	5.67 × 10^−3^	2.49	*
Within-group	22,897.5	25	915.9				
Total	45,167.0	31					

## Data Availability

The original contributions presented in this study are included in the article. Further inquiries can be directed to the corresponding authors.
